# Supplementary material for: Chemical genomics reveals histone deacetylases are required for core regulatory transcription
Source: Nat Commun. 2019 Jul 8;10:3004. doi: 10.1038/s41467-019-11046-7 (PMC6614369; doi:10.1038/s41467-019-11046-7)
Supplement: Supplementary file 1 — Supplementary Information [file 41467_2019_11046_MOESM1_ESM.pdf]

Chemical Genomics Reveals Histone Deacetylases are Required for Core Regulatory Transcription

Gryder, Wu, et al.

Supplementary Figures and Legends

Supplementary Methods, including synthesis of HDAC3 selective inhibitor LW3.

Supplementary Tables

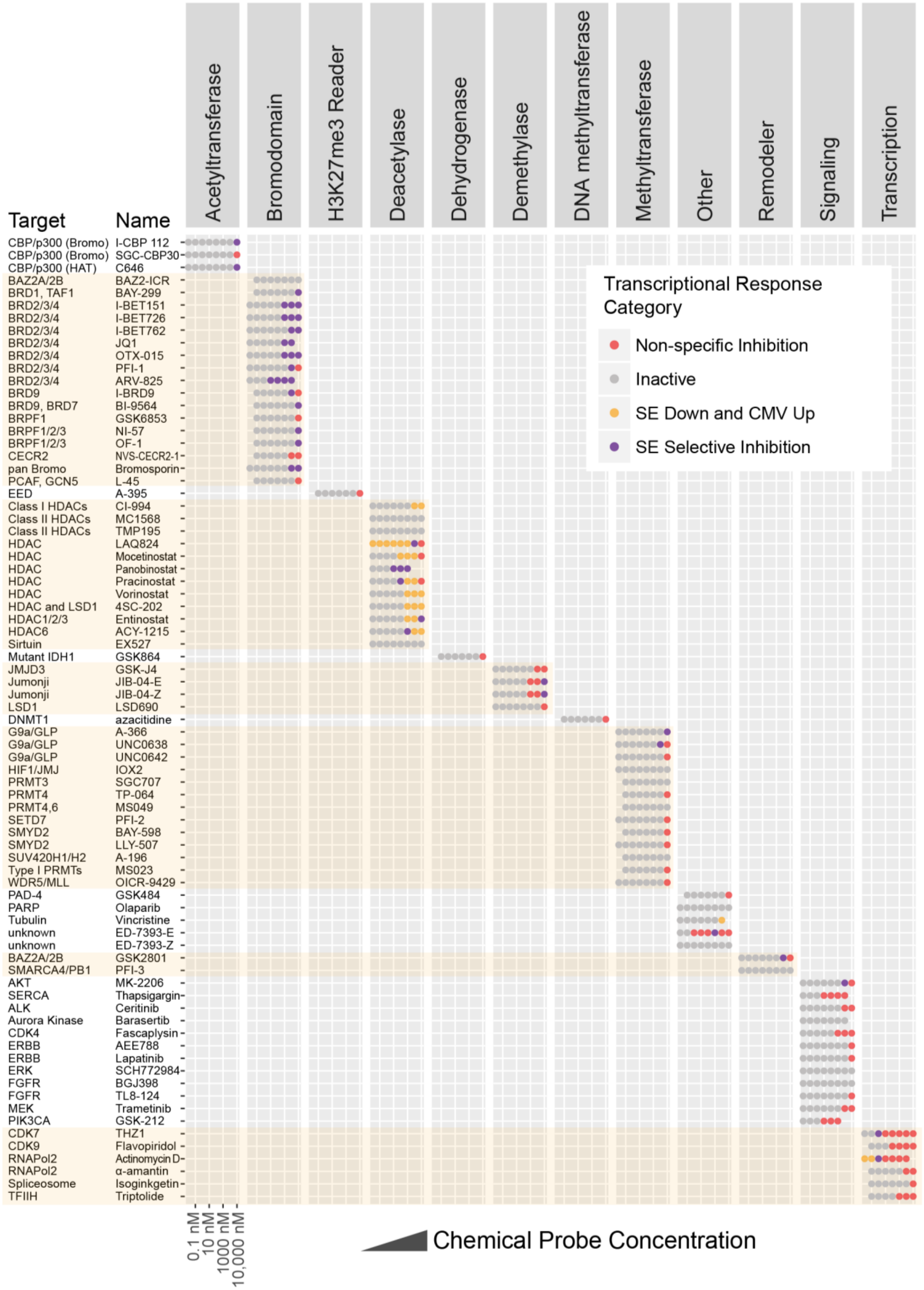

Supplemental Figure 1. Chemical screen for core regulatory transcription

Summary of epigenetic focused SE screen. Each dot represents quadruplicate experiments at the indicated dose of the indicated drug. Known targets and drug names are given in the column to the left, and are sorted by category. Color indicates class as demarcated by the legend.

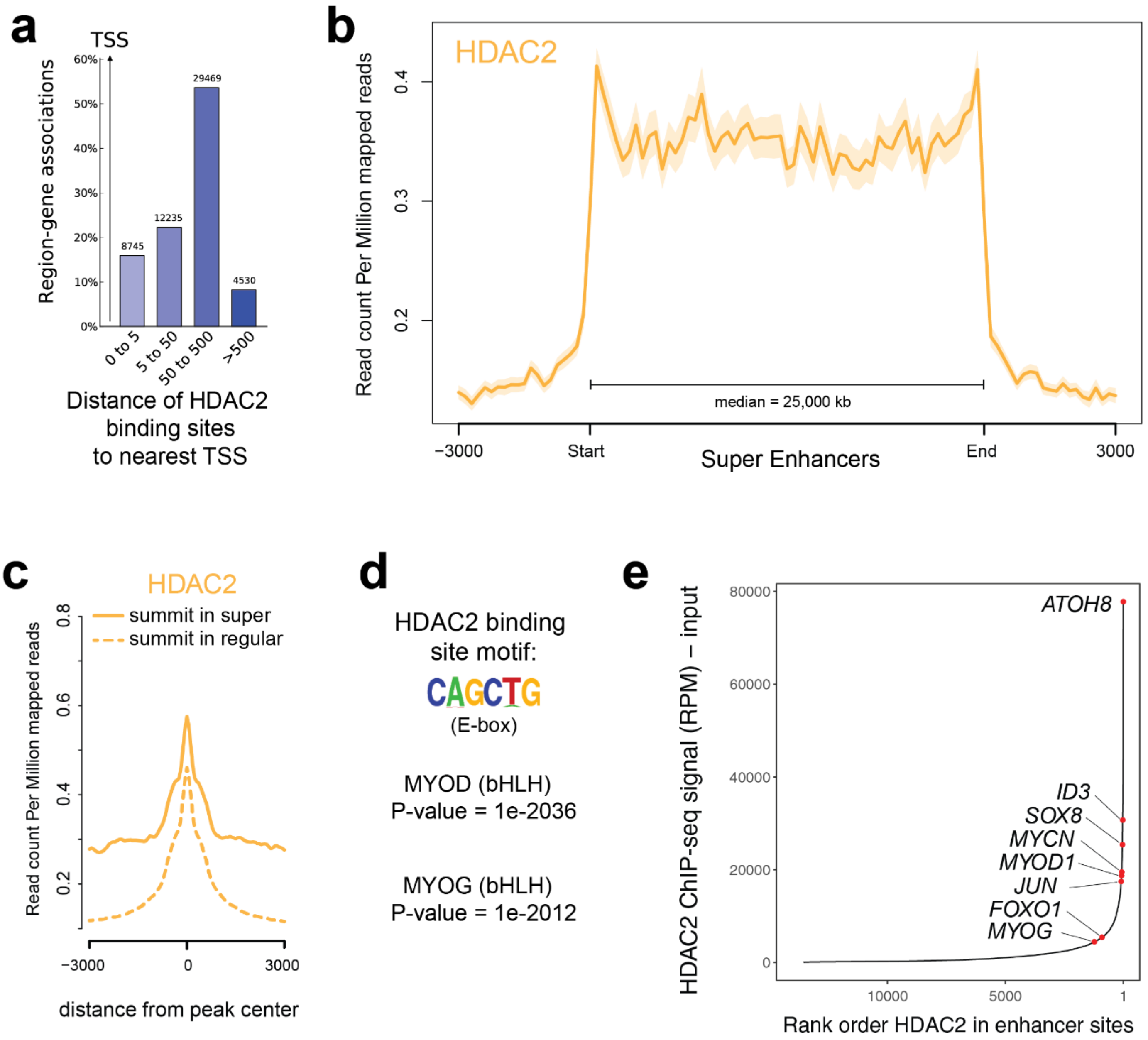

Supplemental Figure 2. Genomic distribution of HDAC2 in Rhabdomyosarcoma cells

(a) Distribution of HDAC2 ChIP-seq peak distances from the nearest TSS, in kilobases.

(b) HDAC2 ChIP-seq profile at super enhancers.

(c) Individual HDAC2 densities for constituent peaks found within enhancers of either super or regular architecture.

(d) Motif enrichment at HDAC2 sites in FP-RMS cell line RH4.

(e) Asymmetric distribution of HDAC2 at genomic enhancer sites, clusters using the same algorithm used more routinely for H3K27ac (ROSE).

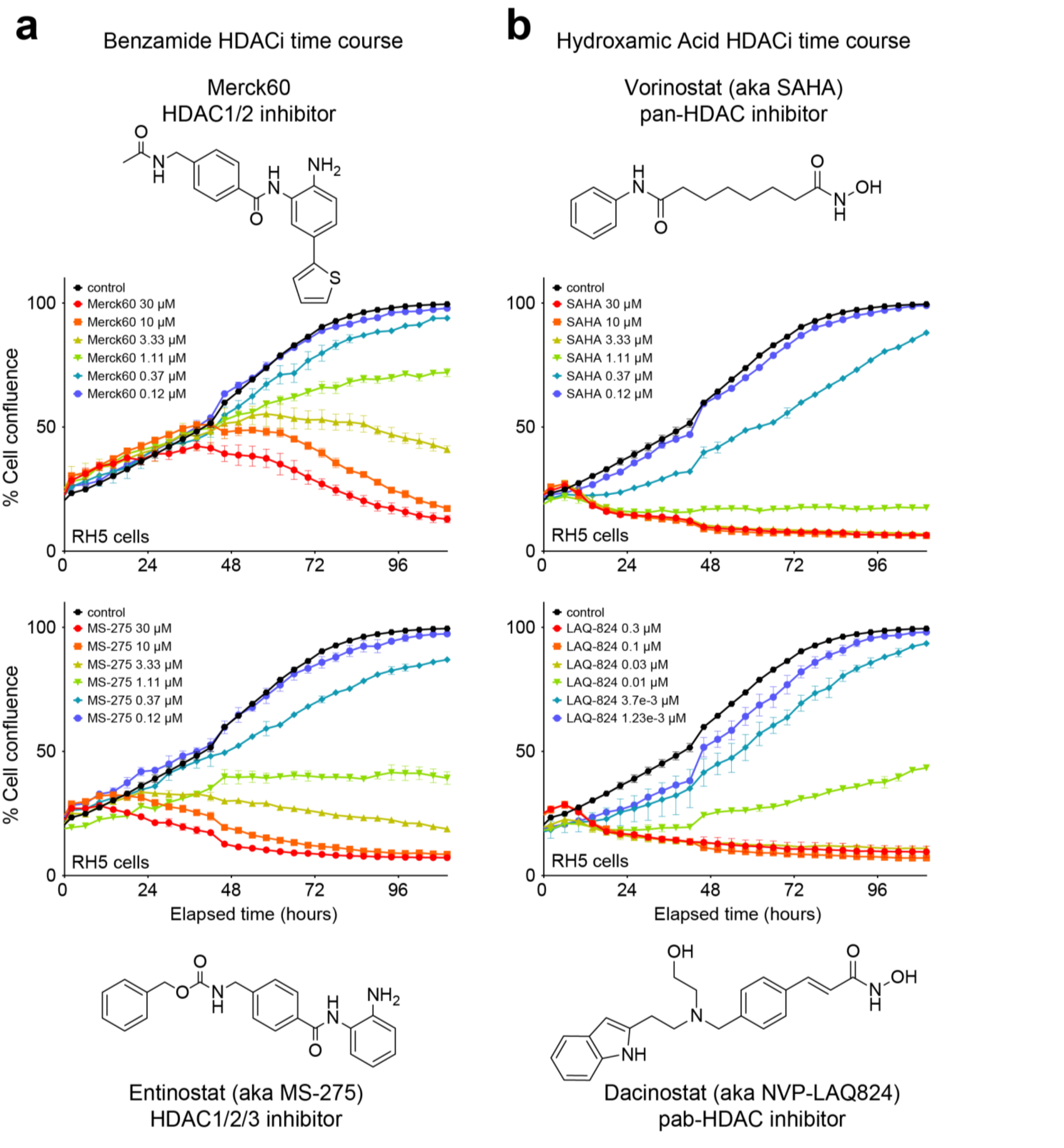

Supplemental Figure 3. Time course of rhabdomyosarcoma cell growth inhibition with HDAC inhibitors

(a) Time course of cell confluence (measure by phase contrast images of RH5 cells every 4 hours) for Benzamide inhibitors Merck60 (selective for HDAC1/2) and Entinostat (selective for HDAC1/2/3).

(b) Same as (a), but treated with Hydroxamic acid based pan-HDAC inhibitors SAHA (Vorinostat, above) and LAQ824 (Dacinostat).

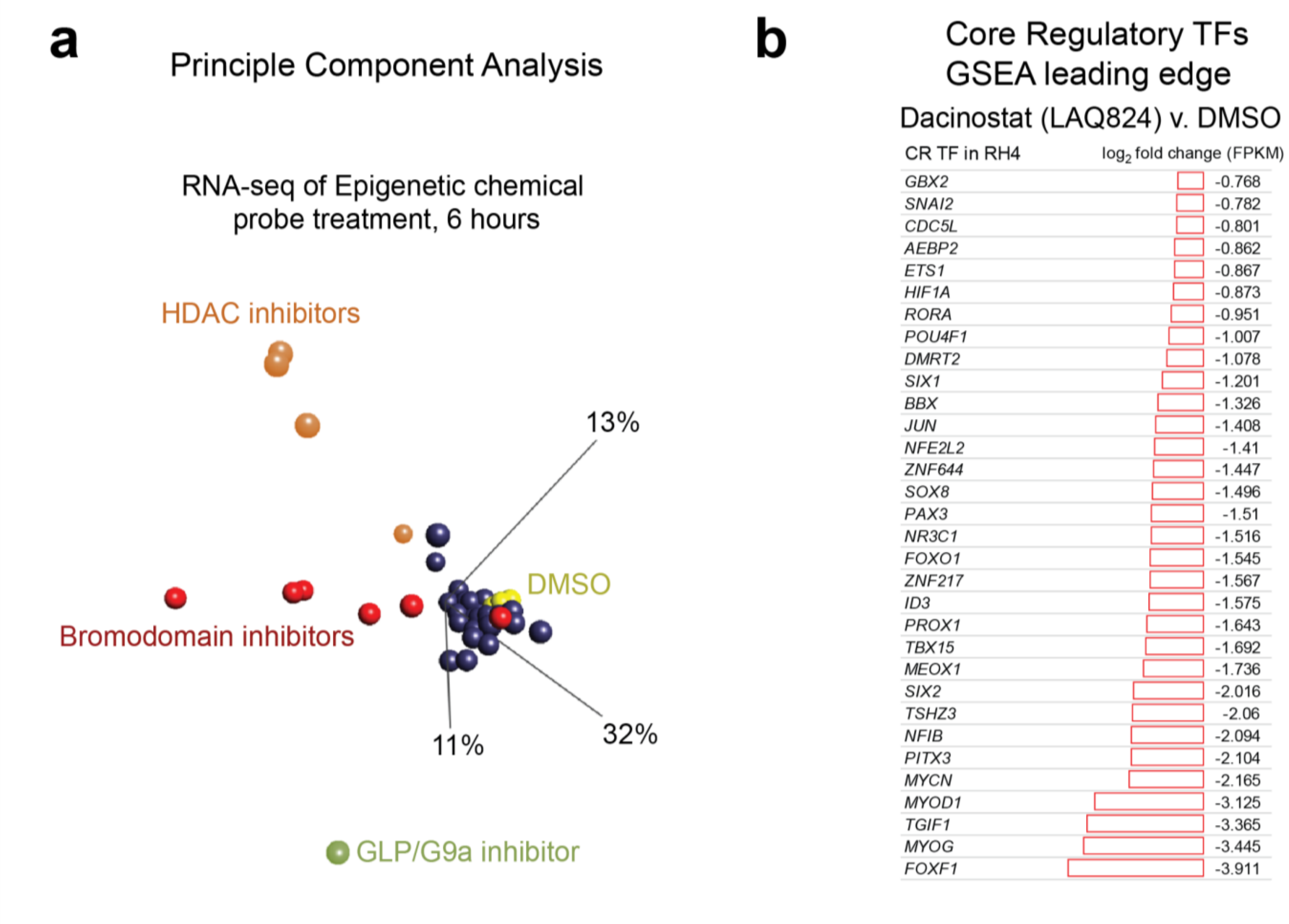

Supplemental Figure 4. RNA-seq after epigenetic chemical probe treatment in Rhabdomyosarcoma cells

(a) Principle component analysis of RNA-seq after 6 hours of drug treatment in RH41 cells. HDAC inhibitors are colored orange, Bromodomain inhibitors colored red, and a GLP/G9A inhibitor is colored green. Most epigenetic chemical probes (dark blue) clustered near control treatment DMSO (yellow).

(b) Example leaded edge of core regulatory TFs downregulated by HDAC inhibitor LAQ824, also known clinically as Dacinostat.

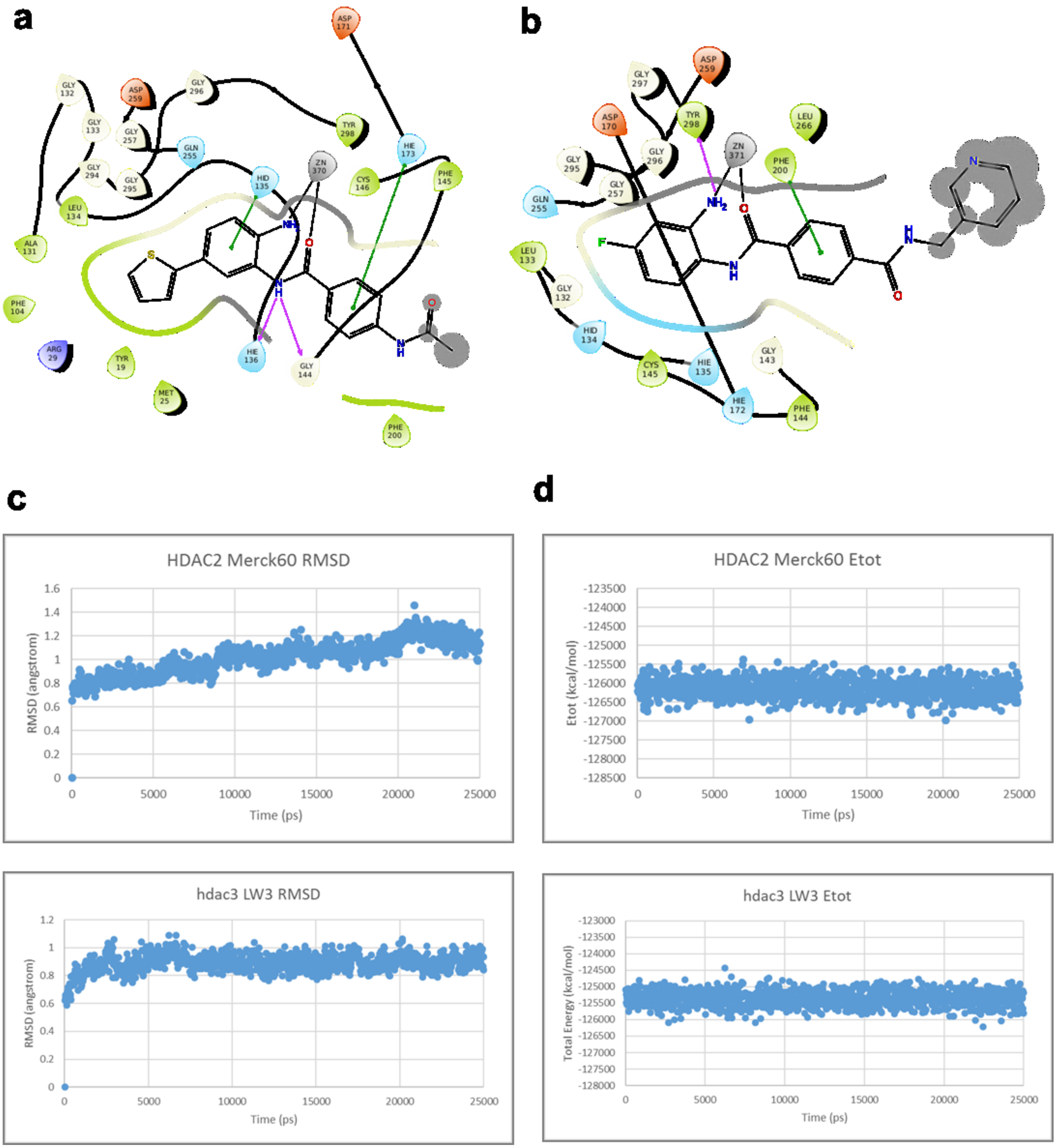

Supplemental Figure 5. Molecular dynamics simulations for Merck60 and LW3 in HDACs

(a, b) Interaction diagrams for Merck60/HDAC2 and LW3/HDAC3 complexes

(c, d) plots of RMSD and total energy showing convergence of MD simulations

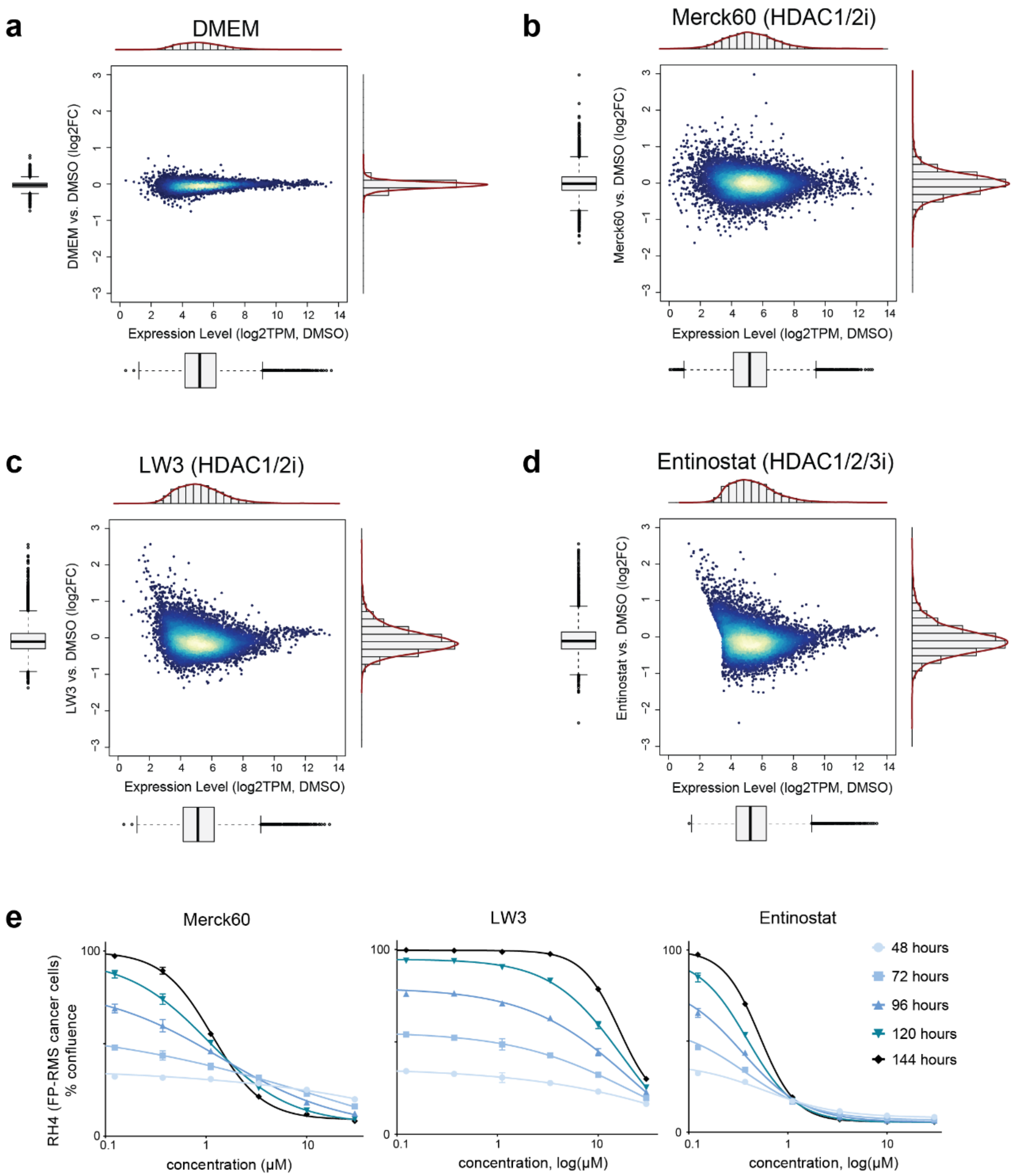

Supplemental Figure 6. Effect of HDAC isoform 1/2/3, 1/2, or 3 selective inhibitors on global transcription and proliferation in RH4 cells

(a-d) MA plots of gene expression changes in RH4 cells treated with the DMEM, Merck60, LW3 or Entinostat for 6 hours (compared to DMSO). Histograms and boxplots summarize the expression distribution shown in the scatter plot.

(e) Proliferation of RH4 cells treated with Merck60, LW3 or Entinostat. Confluence was measured using image analysis of phase contrast images, taken with the IncuCyte ZOOM.

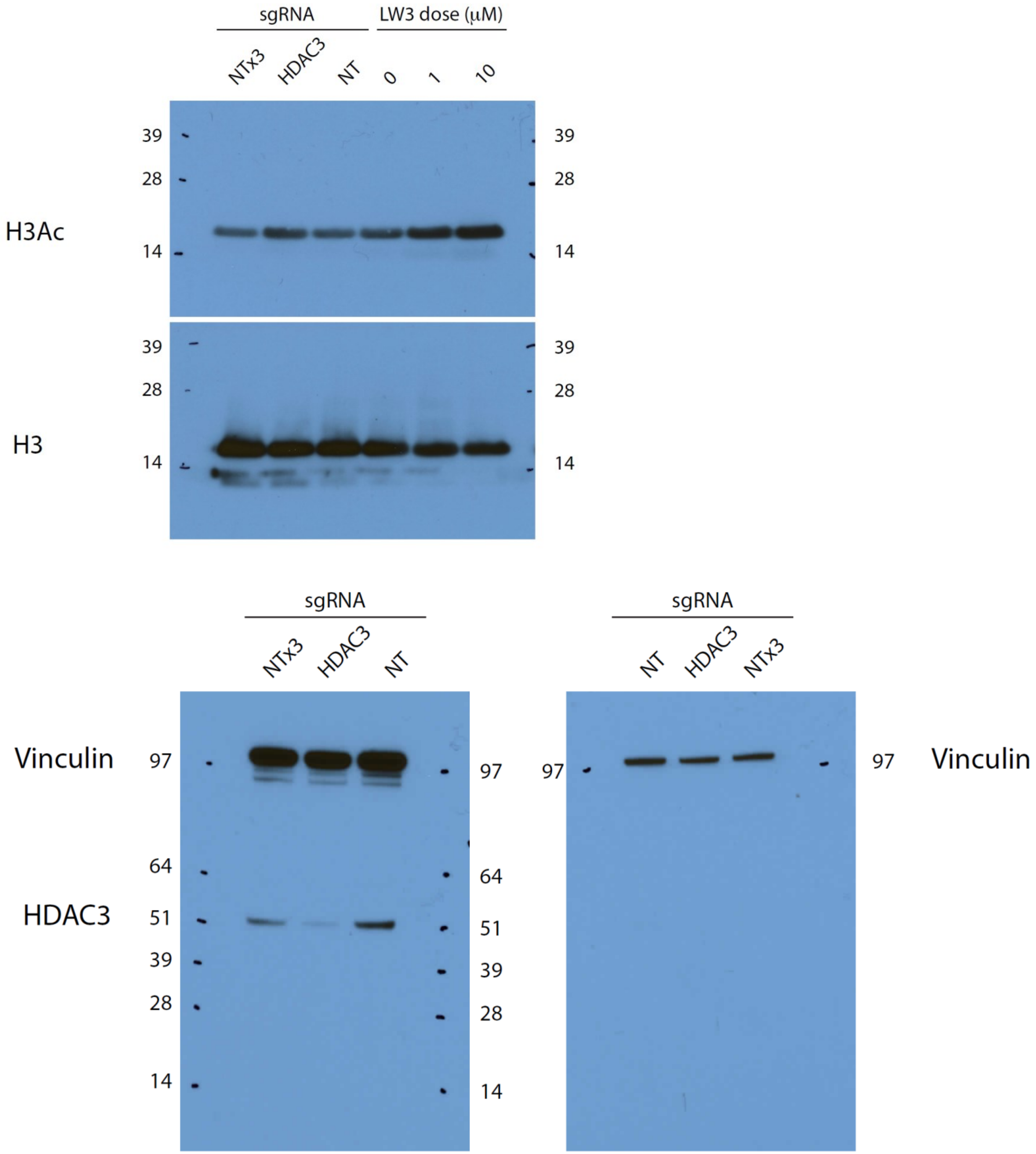

Supplemental Figure 7. Uncropped versions of western blots in Figure 5e, with size markers indicated.

Supplemental Figure 8. Synthetic strategy to obtain HDAC3 selective inhibitor LW3:

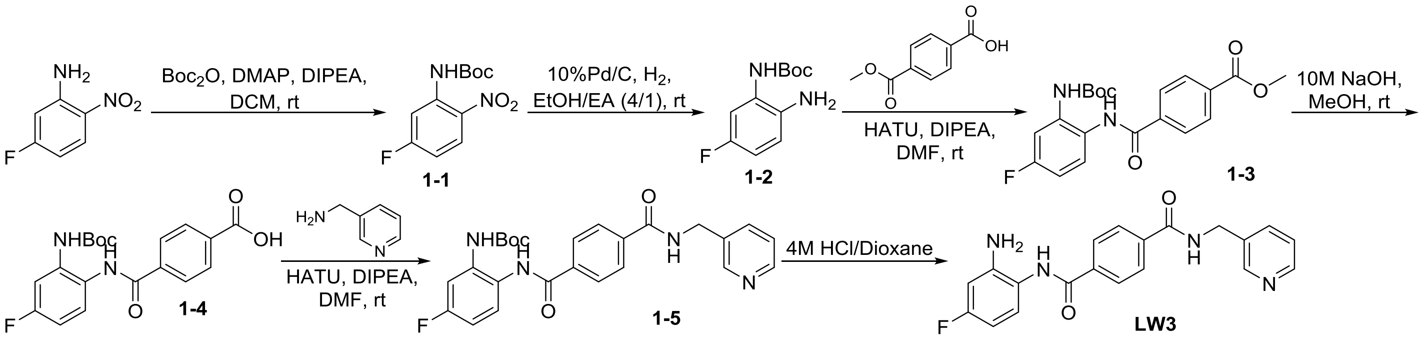

Supplementary Methods

(1) *tert*-Butyl (5-fluoro-2-nitrophenyl)carbamate:

To a solution of 5-fluoro-2-nitroaniline (4.5 g, 28.8 mmol), DMAP (360 mg, 2.95 mmol), and DIPEA (2.5 mL) in anhydrous DCM (60 mL) was added Boc_2O (6.5 g, 29.8 mmol) in portions at room temperature. The reaction mixture was stirred at room temperature for overnight, and purified via silica gel column purification (Ethyl acetate/Hexane: 1/4, R_f = 0.7) to afford **1-1** (6 g, 82% yield) as a yellow solid. MS: m/z $(M+1)^+$: 257.20.

(2) *tert*-Butyl (2-amino-5-fluorophenyl)carbamate:

The solution of **1-1** (6 g, 23.4 mmol) in a mixed solvent of ethanol (120 mL) and ethyl acetate (30 mL) was purged with N_2 3 times followed by the addition of 10% Pd/C (100 mg). The resulting mixture was purged with H_2 3 times and stirred at room temperature under H_2 atmosphere for 4 hours. After filtration, the filtrate was concentrated in vacuo to afford **1-2** as a white solid (5.2 g) MS: m/z $(M+1)^+$: 227.25. ^{1}H NMR (MeOD-d_4, 500 MHz): δ 7.12 (d, J = 8.5 Hz, 1H), 6.79 (dd, J = 8.5 Hz, 5.0 Hz, 1H), 6.70 (td, J = 8.5 Hz, 3.0 Hz, 1H), 1.53 (s, 9H). ^{13}C NMR (MeOD-d_4, 500 MHz): δ 156.98, 155.12, 154.49, 117.52, 117.45, 111.22, 111.04, 79.92, 27.26.

(3) Methyl 4-((2-((*tert*-butoxycarbonyl)amino)-4-fluorophenyl)carbamoyl)benzoate:

To a solution of **1-2** (750 mg, 3.32 mmol), 4-(methoxycarbonyl)benzoic acid (600 mg, 3.33 mmol) and HATU (1.26 g, 3.33 mmol) in DMF (7 mL)was added DIPEA (1.5 mL, 8.44 mmol). The reaction mixture was stirred at room temperature for 30 minutes, and diluted with ethyl acetate (50 mL). The resulting mixture was washed with H_2O (20 mL x 3), and brine (30 mL). The organic phase was dried over anhydrous Na_2SO_4, and filtered. The filtrate was concentrated in vacuo, and the residue was purified via silica gel chromatography to afford **1-3** as a white solid (1.22 g, 94% yield). MS: m/z $(M+H)^+$: 388.12.

(4) 4-((2-((*tert*-butoxycarbonyl)amino)-4-fluorophenyl)carbamoyl)benzoic acid:

To a solution of **1-3** (1.22 g, 3.14 mmol) in MeOH (20 mL) was added 10M NaOH (2 mL). The resulting mixture was stirred at room temperature overnight. After removal of MeOH in vacuo, the residue was acidified to pH ~5 with 6M HCl. The white solid was precipitated, collected by filtration and dried in vacuo overnight to afford **1-4** (1.06 g, 90% yield). MS: m/z $(M+1)^+$: 374.13. ^{1}H NMR (MeOD-d_4, 500 MHz): δ 8.17 (d, J = 8.5 Hz, 2H), 8.07 (d, J = 8.5 Hz, 2H), 7.51 (dd, J = 9.0 Hz, 5.5 Hz, 1H), 7.46 (dd, J = 10.5 Hz, 3.0 Hz, 1H), 6.95 (td, J = 8.5 Hz, 3.0

Hz, 1H), 1.51 (s, 9H). ^{13}C NMR (MeOD-d_4, 500 MHz): δ 167.50, 166.70, 161.86, 159.93, 154.06, 137.82, 133.89, 129.51, 127.88, 127.81, 127.45, 110.67, 110.48, 80.63, 27.13.

(5) *tert*-butyl (5-fluoro-2-(4-((pyridin-3-ylmethyl)carbamoyl)benzamido)phenyl)carbamate:

To the solution of **1-4** (50 mg, 0.134 mmol) and pyridin-3-ylmethanamine (18 μL, 0.180 mmol), and HATU (55 mg, 0.140 mmol) in DMF (0.4 mL) was added DIPEA (65 uL, 0.707 mmol) at room temperature. The mixture was stirred at room temperature for 80 minutes and purified *via* HPLC (0.1% TFA/MeCN) to afford **1-5** as a white solid (60 mg, 95% yield). MS: m/z $(M+1)^+$: 465.21. ^1H NMR (MeOD-d_4, 500 MHz): δ 8.80 (s, 1H), 8.68 (d, J = 5.5 Hz, 1H), 8.32 (d, J = 8.0 Hz, 1H), 7.98 (d, J = 8.5 Hz, 2H), 7.96 (t, J = 10.5 Hz, 1H), 7.92 (d, J = 8.5 Hz, 2H), 7.40 (dd, J = 8.5 Hz, 5.5 Hz, 1H), 7.31 (dd, J = 10.5 Hz, 3.0 Hz, 1H), 6.83 (td, J = 8.5 Hz, 3.0 Hz, 1H), 4.69 (s, 2H), 1.39 (s, 9H). ^{13}C NMR (MeOD-d_4, 500 MHz): δ 171.67, 168.06, 166.46, 161.84, 159.90, 154.12, 145.35, 141.07, 140.57, 139.84, 137.08, 136.46, 127.70, 127.42, 127.02, 110.80, 110.62, 80.69, 40.41, 27.24.

(6) *N*^{1}-(2-amino-4-fluorophenyl)-*N*^{4}-(pyridin-3-ylmethyl)terephthalamide:

The 4M solution of HCl in dioxane (0.5 mL) was added to **1-5** (50 mg). The reaction mixture was stirred at room temperature overnight, concentrated and dried *in vacuo* to afford **LW3** as a white solid (60 mg). MS: m/z $(M+H)^+$: 365.24. ^1H NMR (MeOD-d_4, 500 MHz): δ 8.96 (s, 1H), 8.83 (d, J = 6.0 Hz, 1H), 8.72 (d, J = 8.5 Hz, 1H), 8.19 (d, J = 8.5 Hz, 2H), 8.14 (dd, J = 8.5 Hz, 6.0 Hz, 1H), 8.09 (d, J = 9.0 Hz, 2H), 7.51 (dd, J = 8.0 Hz, 5.5 Hz, 1H), 7.29-7.26 (m, 2H), 4.83 (s, 2H). ^{13}C NMR (MeOD-d_4, 500 MHz): δ 168.00, 166.86, 162.01, 146.00, 140.64, 140.12, 136.76, 136.15, 128.40, 128.32, 128.12, 127.45, 127.22, 115.13, 114.97, 110.56, 110.35, 40.35.

Supplementary Table 1. Epigenetic Chemical Probes

**Drug Name**	**Source**	**Target**
4SC-202	DTP	HDAC and LSD1
A-196	SGC - Peter Brown	SUV420H1/H2
A-366	SGC - Peter Brown	G9a/GLP
A-366	SGC - Peter Brown	G9a/GLP
A-395	SGC - Peter Brown	EED
Actinomycin D	DTP	RNAPol2
ACY-1215	DTP	HDAC6
AEE788	Selleckchem	ERBB
alpha-amantin	DTP	RNAPol2
ARV-825	DTP	BRD4
azacitidine	DTP	DNMT1
Barasertib	Sam Li	Aurora Kinase
BAY-299	SGC - Peter Brown	BRD1, TAF1
BAY-598	SGC - Peter Brown	SMYD2
BAZ2-ICR	SGC - Peter Brown	BAZ2A, BAZ2B
BGJ398	Mari Yohe	FGFR
BI-9564	SGC - Peter Brown	BRD9, BRD7
Bromosporin	SGC - Peter Brown	pan Bromo
C646	SGC - Peter Brown	EP300
CI-994	SGC - Peter Brown	Class I HDACs
Cpd-50	Jay Bradner & Jun Qi	pan-JMJD
EX 527 (Selisistat)	Selleckchem	Sirtuin
Fascaplysin	Santa Cruz	CDK4
Flavopiridol	DTP	CDK9
GSK2801	SGC - Peter Brown	BAZ2A/2B
GSK2801	SGC - Peter Brown	BAZ2A/2B
GSK484	SGC - Peter Brown	PAD-4
GSK6853	SGC - Peter Brown	BRPF1
GSK864	SGC - Peter Brown	Mutant IDH1
GSK-J4	Jay Bradner & Jun Qi	JMJD3
GSK-LSD1	SGC - Peter Brown	LSD1
I-Bet-151	DTP	BRD2/3/4
I-Bet-726	DTP	BRD2/3/4
I-Bet-762	DTP	BRD2/3/4
I-BRD9	SGC - Peter Brown	BRD9
I-CBP 112	SGC - Peter Brown	CREBBP EP300
Isoginkgetin	DTP	Spliceosome
JIB-04-E	Martinez	Jumonji
JIB-04-Z	Martinez	Jumonji
JQ1	Jay Bradner & Jun Qi	BRD2/3/4
LAQ824	SGC - Peter Brown	HDAC
LLY-507	SGC - Peter Brown	SMYD2
LSD-519	Jay Bradner & Jun Qi	LSD1
LSD-690	Jay Bradner & Jun Qi	LSD1

MC1568	Tocris	Class II HDACs
Merck60	Jay Bradner & Jun Qi	HDAC1/2
Mocetinostat	DTP	HDAC
MS023	SGC - Peter Brown	Type I PRMTs
MS049	SGC - Peter Brown	PRMT4,6
MS-275 (Entinostat)	DTP	HDAC1/2/3
NI-57	SGC - Peter Brown	BRPF1/2/3
NP-005584	Girma Woldemichael	pan-HDACi
NVS-CECR2-1	SGC - Peter Brown	CECR2
OF-1	SGC - Peter Brown	BRPF1/2/3
OICR-9429	SGC - Peter Brown	WDR5/MLL
OICR-9429	SGC - Peter Brown	WDR5/MLL
OJI-1	Jay Bradner & Jun Qi	HDAC8
Olaparib	SGC - Peter Brown	PARP
OTX-015	DTP	BRD2/3/4
Panobinostat	DTP; Novartis	HDAC
PFI-1	SGC - Peter Brown	BRD2/3/4
PFI-1	SGC - Peter Brown	BRD2/3/4
PFI-2	SGC - Peter Brown	SETD7
PFI-2	SGC - Peter Brown	SETD7
PFI-3	SGC - Peter Brown	SMARCA bromodomains
PFI-3	SGC - Peter Brown	SMARCA bromodomains
Pracinostat	DTP	pan-HDACs
Quisinostat	DTP	pan-HDACs
R18	Tocris	14.3.3
Rapamycin	Khanna	mTOR
Rigosertib	Selleckchem	microtubules
Romidepsin	Selleckchem	pan-HDACs
SCG-CBP30	SGC - Peter Brown	p300
SCH772984	DTP	ERK
Selumetinib	DTP	MEK
SGC0946	SGC - Peter Brown	DOT1L
SGC707	SGC - Peter Brown	PRMT3
SGC-CBP30	SGC - Peter Brown	CREBBP EP300
Thapsigargin	Santa Cruz	AKT
THZ1	Nat Gray	CDK7
TMP195	Axon Medchem	Class II HDACs
TP-064	SGC - Peter Brown	PRMT4
Trametinib	Selleckchem	MEK
Triptolide	DTP	TFIIH
UNC0638	SGC - Peter Brown	G9a/GLP
UNC0642	SGC - Peter Brown	G9a/GLP
UNC1215	SGC - Peter Brown	L3MBTL3
UNC1999	SGC - Peter Brown	EZH1/2
WT161	Jay Bradner & Jun Qi	HDAC6

Supplementary Table 2. Primers for 4C-seq and ChIP-qPCR

4C-seq	MYOD1 Super Enhancer 4C viewpoint forward primer	AGGTTCTGCGACAGAGTTGG
	MYOD1 Super Enhancer 4C viewpoint reverse primer	GTTTGGGTTTGGCTGGCTTG
	MYOD1 promoter 4C viewpoint forward primer	GGGATTCCTAACCTGGGCAG
	MYOD1 promoter 4C viewpoint reverse primer	AGTTGGATGCTGTGTCCCG
ChIP-qPCR	CTCF site IGF2 SE-proximal, forward primer	CCACTAGATGGCAGTCACAC
	CTCF site IGF2 SE-proximal, reverse primer	GGCCCCTGTTTAGAACAACA
	P3F site at MYOD1 SE, forward primer	CAGAACCATCCCATTCTCCG
	P3F site at MYOD1 SE, reverse primer	GCCTGACCTTGAACGTGAAT
	Negative control gene desert, forward primer	AGGGAGTTTTTATGAGCATTCCA
	Negative control gene desert, reverse primer	AGCAGGTAAAGGTCCATATTTCA

Supplementary Table 3. **REAGENTS AND RESOURCES TABLE**

REAGENT or RESOURCE	SOURCE	IDENTIFIER
Antibodies		
Rabbit polyclonal anti-HDAC2	Abcam	Cat# 7029, RRID:AB_305706
Rabbit polyclonal anti-SOX8	Abcam	Cat# ab104245, RRID:AB_10974591
Rabbit polyclonal anti-H3K27ac	Active Motif	Cat# 39133, RRID:AB_2561016
Rabbit polyclonal anti-HDAC1	Abcam	Cat# 7028, RRID:AB_305705
Rabbit polyclonal anti-YY1	Abcam	Cat# ab109237, RRID:AB_10890662
Mouse monoclonal Anti-Vinculin	Sigma-Aldrich	Cat# V9264, RRID:AB_10603627
Goat Polyclonal anti-mouse IgG-HRP	Santa Cruz Biotechnology	Cat# sc-2005, RRID:AB_631736
Rabbit polyclonal anti-HDAC3	Abcam	Cat# ab7030, RRID:AB_305708
Rabbit polyclonal anti-Histone H3ac (acetyl K9 + K14 + K18 + K23 + K27)	Abcam	Cat# ab47915, RRID:AB_873860
Rabbit polyclonal anti-Histone H3	Cell Signaling Technology	Cat# 9715, RRID:AB_331563
Goat anti-rabbit IgG-HRP	Santa Cruz Biotechnology	Cat# sc-2004, RRID:AB_631746
Chemicals, Peptides, and Recombinant Proteins		
Polyethylenimine, branched PEI 25000	Sigma Aldrich	Cat# 408727
Actinomycin D	Sigma Aldrich	Cat# A1410
List of small molecules screened, related to Figure 2, (Table S1)	This study	N/A
Kits and Commercial Assays		
AmpFLSTR Identifiler PCR Amplification Kit	Life Technologies	Cat# 4322288
Nextera DNA Library Prep Kit	Illumina	Cat# FC-121-1030
MinElute PCR Purification Kit	Qiagen	Cat# 28004
Agencourt AMPure XP	Beckman Coulter	Cat# A63881
ChIP-IT High Sensitivity	Active Motif	Cat# 53040
QIAamp DNA Mini Kit	Qiagen	Cat# 51304
T4 DNA polymerase	NEB	Cat# B02025
Large (Klenow) Fragment	NEB	Cat# M0210L
T4 polynucleotide kinase	NEB	Cat# M0201L
Quick ligation kit	NEB	Cat# M2200L
Phusion master mix	Thermo Fisher Scientific	Cat# F548L
SteadyLite Plus luciferase assay reagent powder	PerkinElmer	Cat# 6066759
TruSeq ChIP Library Prep Kit	Illumina	Cat# IP-202-1012
Dynabead M-280 Streptavidin	Thermo	Cat#: 11205D
ChIP-IT qPCR Analysis Kit	Active Motif	Cat# 53029
NextSeq500 High Output Kit v2 (75 cycles)	Illumina	Cat# FC-404-2005
Nextera XT DNA Library Preparation Kit	Illumina	Cat# FC-131-1024
Deposited Data		
Raw and analyzed data	This paper	GEO: GSE116344
ChIP-seq in rhabdomyosarcoma cells and tumors	Gryder et al 2017	GEO: GSE83728
Topologically Associated Domain boundaries	Rao et al., 2014	GEO: GSE63525

ENCODE blacklisted regions	ENCODE Project Consortium, 2012	http://hgdownload.cse.ucsc.edu/goldenPath/hg19/encodeDCC/wgEncodeMapability/wgEncodeDacMapabilityConsensusExcludable.bed.gz
Experimental Models: Cell Lines		
Human cancer cell lines: RH4, RH41, RH3, RH5	Peter Houghton Lab	http://gsbs.uthscsa.edu/faculty/peter-houghton-ph.d
Recombinant DNA		
pGreenFire1-CMV Positive Control Lentivector	System Biosciences	Cat# TR011PA/VA-1
pGreenFire1-mCMV Negative Control	System Biosciences	Cat# TR010PA/VA-1
Software and Algorithms		
R statistical package drc	Christian Ritz	https://cran.r-project.org/web/packages/drc/drc.pdf
NGSplot (v. 2.63)	Shen Lab	https://github.com/shenlab-sinai/ngsplot
AMBER16	Case et al, 2016	http://ambermd.org
Gaussian09	Gaussian Inc.	http://gaussian.com
Pymol	Schrödinger Inc.	http://schrodinger.com
Graphpad Prism	Graphpad Software	https://www.graphpad.com/scientific-software/prism/
ROSE2	Charles Lin Lab	https://github.com/linlabbcm/rose2
Coltron	Lin et al, 2016	https://pypi.org/project/coltron/
Bamliquidator version 1.3.4	John DiMatteo	https://github.com/BradnerLab/pipeline/wiki/bamliquidator
GSEA software version 2.2.0	Subramanian et al, 2005	https://software.broadinstitute.org/gsea/
Bedtools version 2.27.1	Quinlan and Hall, 2010	http://bedtools.readthedocs.io/en/latest/
RNA-seq Plotting R Scripts	This paper	https://github.com/GryderArt/VisualizeRNAseq
