# Supplementary material for: Chemical genomics reveals histone deacetylases are required for core regulatory transcription
Source: Nat Commun. 2019 Jul 8;10:3004. doi: 10.1038/s41467-019-11046-7 (PMC6614369; doi:10.1038/s41467-019-11046-7)
Supplement: Supplementary file 3 — Reporting Summary [file 41467_2019_11046_MOESM3_ESM.pdf]

nature research

Corresponding author(s): Jun Qi and Javed Khan

Reporting Summary

Nature Research wishes to improve the reproducibility of the work that we publish. This form provides structure for consistency and transparency in reporting. For further information on Nature Research policies, see Authors & Referees and the Editorial Policy Checklist.

Statistical parameters

When statistical analyses are reported, confirm that the following items are present in the relevant location (e.g. figure legend, table legend, main text, or Methods section).

n/a	Confirmed	
☐	☒	The exact sample size (*n*) for each experimental group/condition, given as a discrete number and unit of measurement
☐	☒	An indication of whether measurements were taken from distinct samples or whether the same sample was measured repeatedly
☐	☒	The statistical test(s) used AND whether they are one- or two-sided<br>*Only common tests should be described solely by name; describe more complex techniques in the Methods section.*
☒	☐	A description of all covariates tested
☒	☐	A description of any assumptions or corrections, such as tests of normality and adjustment for multiple comparisons
☐	☒	A full description of the statistics including central tendency (e.g. means) or other basic estimates (e.g. regression coefficient) AND variation (e.g. standard deviation) or associated estimates of uncertainty (e.g. confidence intervals)
☐	☒	For null hypothesis testing, the test statistic (e.g. *F*, *t*, *r*) with confidence intervals, effect sizes, degrees of freedom and *P* value noted<br>*Give P values as exact values whenever suitable.*
☒	☐	For Bayesian analysis, information on the choice of priors and Markov chain Monte Carlo settings
☐	☒	For hierarchical and complex designs, identification of the appropriate level for tests and full reporting of outcomes
☐	☒	Estimates of effect sizes (e.g. Cohen's *d*, Pearson's *r*), indicating how they were calculated
☐	☒	Clearly defined error bars<br>*State explicitly what error bars represent (e.g. SD, SE, CI)*

Our web collection on statistics for biologists may be useful.

Software and code

Policy information about availability of computer code

Data collection	Data collection was performed with Illumina's NextSeq 550 system.
Data analysis	Software and Algorithms<br>R statistical package drc Christian Ritz https://cran.r-project.org/web/packages/drc/drc.pdf<br>NGSplot (v. 2.63) Shen Lab https://github.com/shenlab-sinai/ngsplot<br>MACS (v 2.1.1.20160309) Zhang et al 2008 https://github.com/taoliu/MACS<br>BWA (v 0.7.17) Li and Durbin, 2009 http://bio-bwa.sourceforge.net/<br>HOMER (Hypergeometric Optimization of Motif EnRichment) version 4.9.1 Heinz et al, 2010 http://homer.ucsd.edu/homer/index.html<br>Graphpad Prism Graphpad Software https://www.graphpad.com/scientific-software/prism/<br>ROSE2 Charles Lin Lab https://github.com/linlabbcm/rose2<br>Coltron Lin et al, 2016 https://pypi.org/project/coltron/<br>Bamliquidator version 1.3.4 John DiMatteo https://github.com/BradnerLab/pipeline/wiki/bamliquidator<br>GSEA software version 2.2.0 Subramanian et al, 2005 https://software.broadinstitute.org/gsea/<br>Bedtools version 2.27.1 Quinlan and Hall, 2010 http://bedtools.readthedocs.io/en/latest/

For manuscripts utilizing custom algorithms or software that are central to the research but not yet described in published literature, software must be made available to editors/reviewers upon request. We strongly encourage code deposition in a community repository (e.g. GitHub). See the Nature Research guidelines for submitting code & software for further information.

Data

Policy information about availability of data

All manuscripts must include a data availability statement. This statement should provide the following information, where applicable:

- Accession codes, unique identifiers, or web links for publicly available datasets
- A list of figures that have associated raw data
- A description of any restrictions on data availability

The data reported herein is made publicly available through the Gene Expression Omnibus (https://www.ncbi.nlm.nih.gov/geo/). The GEO accession number for all ChIP-seq and RNA-seq data is GSE116344.

Field-specific reporting

Please select the best fit for your research. If you are not sure, read the appropriate sections before making your selection.

☒ Life sciences ☐ Behavioural & social sciences ☐ Ecological, evolutionary & environmental sciences

For a reference copy of the document with all sections, see nature.com/authors/policies/ReportingSummary-flat.pdf

Life sciences study design

All studies must disclose on these points even when the disclosure is negative.

Sample size	For each ChIP-seq and RNA-seq experiment, approximately 30 million uniquely mapped reads were generated.
Data exclusions	Regions of the genome on the ENCODE blacklist (comprised of, for instance, highly repetitive regions) were excluded from called peaks prior to all downstream analysis (ie, in ChIP-seq data sets)
Replication	For luciferase screening, each molecule (n = 63,000) we tested in duplicate initially at one concentration (10 uM). Validation was performed by dose response of top hits (n = 573) in technical quadruplicate. For luciferase screening of epigenetic drug panel, we performed dose response with technical quadruplicates and biological duplicates (only first biological replicate is shown in figures).<br>For cell growth of HDAC inhibitors (n = 14 inhibitors) we tested RMS cell lines (n = 8) and treated in technical duplicate and dose response. Measurements for confluence were taken every 4 hours.<br>For RNA with each small molecule tested, multiple concentrations (IC50 and IC90) were used and data analysis (ie, GSEA) was performed by treating these two concentrations as duplicates.<br>For ChIP-seq of individual factors, we have only presented one experimental dataset per factor, although in many cases we have performed these experiments in biological duplicate or triplicate, or across multiple cell lines with multiple antibodies (data not presented).
Randomization	This work involved no animal studies or randomized clinical trial data. All RMS patients whose samples were obtained were not the subject of randomization at the time of sampling.
Blinding	Investigation was done without blinding, and epigenetic and transcriptomic analysis of primary tumors was done with diagnosis knowledge in hand.

Reporting for specific materials, systems and methods

Materials & experimental systems

n/a	Involved in the study
☐	☒ Unique biological materials
☐	☒ Antibodies
☐	☒ Eukaryotic cell lines
☒	☐ Palaeontology
☒	☐ Animals and other organisms
☒	☐ Human research participants

Methods

n/a	Involved in the study
☐	☒ ChIP-seq
☒	☐ Flow cytometry
☒	☐ MRI-based neuroimaging

Unique biological materials

Policy information about availability of materials

Obtaining unique materials	All cell lines and reagents described in the methods section are either commercially available or available upon request to the authors. Materials not available to the public include the rare and limited primary tumor samples used to generate data herein.

Antibodies

Antibodies used	Rabbit polyclonal anti-HDAC2 Abcam Cat# 7029, RRID:AB_305706<br>Rabbit polyclonal anti-SOX8 Abcam Cat# ab104245, RRID:AB_10974591<br>Rabbit polyclonal anti-H3K27ac Active Motif Cat# 39133, RRID:AB_2561016<br>Rabbit polyclonal anti-HDAC1 Abcam Cat# 7028, RRID:AB_305705<br>Rabbit polyclonal anti-YY1 Abcam Cat# ab109237, RRID:AB_10890662<br>Rabbit polyclonal anti-HDAC3, Abcam Cat# ab7030, RRID:AB_305708<br>Rabbit polyclonal anti-Histone H3ac (acetyl K9 + K14 + K18 + K23 + K27) Abcam Cat# ab47915, RRID:AB_873860<br>Rabbit polyclonal anti-Histone H3 Cell Signaling Technology Cat# 9715, RRID:AB_331563<br>Goat anti-rabbit IgG-HRP Santa Cruz Biotechnology Cat# sc-2004, RRID:AB_631746
Validation	Antibody validations are available on these websites: www.abcam.com, www.scbt.com/scbt and www.activemotif.com<br>In the case of the antibodies for SOX8, which were previously validated by western blot but not validated for ChIP-seq, we evaluated enrichments at predicted SOX binding site in RMS cells by ChIP-qPCR and reviewed the products here: https://www.abcam.com/SOX8-antibody-ab104245/reviews/57941

Eukaryotic cell lines

Policy information about cell lines

Cell line source(s)	Human cancer cell lines: RH4, RH41, RH3, RH5 Peter Houghton Lab http://gsbs.uthscsa.edu/faculty/peter-houghton-ph.d<br>SCMC line is from Dr. Janet Shipley, RD, SMS-CTR and Birch from Dr. Lee Helman.
Authentication	Validation was performed by DNA fingerprinting AmpFlSTR® Identifiler® PCR Amplification Kit (Catalog Number 4322288) by Life Technologies. Additional validation was done by RNA-seq comparison to historic cell line RNA-seq data.
Mycoplasma contamination	Cell lines were tested for mycoplasma frequently within one or two passages of each experiment.
Commonly misidentified lines<br>(See ICLAC register)	No misidentified cell lines were used in this study.

ChIP-seq

Data deposition

☒ Confirm that both raw and final processed data have been deposited in a public database such as GEO.

☒ Confirm that you have deposited or provided access to graph files (e.g. BED files) for the called peaks.

Data access links<br>*May remain private before publication.*	To review ChIP-seq and RNA-seq data, use GEO accession GSE116344:<br>Go to https://www.ncbi.nlm.nih.gov/geo/query/acc.cgi?acc=GSE116344<br>Enter token incneucolzutdgj into the box
Files in database submission	Rh4_DMSO_T_HCK52BGXY.gene.TPM.txt<br>Rh4_N1302_T_HCK52BGXY.gene.TPM.txt<br>Rh4_PLX1_T_HCK52BGXY.gene.TPM.txt<br>Rh4_PLX2_T_HCK52BGXY.gene.TPM.txt<br>RH41Bromosporinhigh_T_C5JW5ACXX.gene.TPM.txt<br>RH41Bromosporinlow_T_C5JW5ACXX.gene.TPM.txt<br>RH41Cpd50high_T_C4JM1ACXX.gene.TPM.txt<br>RH41Cpd50low_T_C4JM1ACXX.gene.TPM.txt<br>RH41DMSOhigh_T_C4JM1ACXX.gene.TPM.txt<br>RH41DMSOhigh_T_H144EBGXX.gene.TPM.txt<br>RH41DMSOlow_T_C4JM1ACXX.gene.TPM.txt<br>RH41DMSOlow_T_H144EBGXX.gene.TPM.txt<br>RH41EZ-005high_T_C5JW5ACXX.gene.TPM.txt<br>RH41EZ-005low_T_C5JW5ACXX.gene.TPM.txt<br>RH41GSKJ4high_T_C4JM1ACXX.gene.TPM.txt<br>RH41GSKJ4low_T_C4JM1ACXX.gene.TPM.txt<br>RH41GSKLSD1high_T_H0VWNAGXX.gene.TPM.txt<br>RH41GSKLSD1low_T_H0VWNAGXX.gene.TPM.txt<br>RH41JQ1high_T_C4JM1ACXX.gene.TPM.txt

RH41JQ1low_T_C4JM1ACXX.gene.TPM.txt
RH41LAQ824high_T_H144EBGXX.gene.TPM.txt
RH41LAQ824low_T_H144EBGXX.gene.TPM.txt
RH41LSD519high_T_C5JW5ACXX.gene.TPM.txt
RH41LSD519low_T_C5JW5ACXX.gene.TPM.txt
RH41LSD690high_T_C5JW5ACXX.gene.TPM.txt
RH41LSD690low_T_C5JW5ACXX.gene.TPM.txt
RH41MC1568high_T_H144EBGXX.gene.TPM.txt
RH41MC1568low_T_H144EBGXX.gene.TPM.txt
RH41Merck60high_T_C5JW5ACXX.gene.TPM.txt
RH41Merck60low_T_C5JW5ACXX.gene.TPM.txt
RH41OJI1high_T_C5JW5ACXX.gene.TPM.txt
RH41OJI1low_T_C5JW5ACXX.gene.TPM.txt
RH41PFI2high_T_C5JW5ACXX.gene.TPM.txt
RH41PFI2low_T_C5JW5ACXX.gene.TPM.txt
RH41PFI3high_T_C5JW5ACXX.gene.TPM.txt
RH41PFI3low_T_C5JW5ACXX.gene.TPM.txt
RH41SGC0946high_T_C5JW5ACXX.gene.TPM.txt
RH41SGC0946low_T_C5JW5ACXX.gene.TPM.txt
RH41SGCCBP30high_T_C5JW5ACXX.gene.TPM.txt
RH41SGCCBP30low_T_C5JW5ACXX.gene.TPM.txt
RH41Tramhigh_T_C5JW5ACXX.gene.TPM.txt
RH41Tramlow_T_C5JW5ACXX.gene.TPM.txt
RH41UNC0638high_T_C5JW5ACXX.gene.TPM.txt
RH41UNC0638low_T_C5JW5ACXX.gene.TPM.txt
RH41UNC1215high_T_H0VWNAGXX.gene.TPM.txt
RH41UNC1215low_T_H0VWNAGXX.gene.TPM.txt
RH41UNC1999high_T_C5JW5ACXX.gene.TPM.txt
RH41UNC1999low_T_C5JW5ACXX.gene.TPM.txt
RH41WT161high_T_C5JW5ACXX.gene.TPM.txt
RH41WT161low_T_C5JW5ACXX.gene.TPM.txt
RH4_D1_T_HVNVFBGX2.gene.TPM.txt
RH4_D24_T_HVNVFBGX2.gene.TPM.txt
RH4_D6_T_HVNVFBGX2.gene.TPM.txt
RH4_ENT1_T_HVNVFBGX2.gene.TPM.txt
RH4_ENT24_T_HVNVFBGX2.gene.TPM.txt
RH4_ENT6_T_HVNVFBGX2.gene.TPM.txt
Sample_RH41Bromosporinhigh_T_C5JW5ACXX_R1.fastq.gz
Sample_RH41Bromosporinhigh_T_C5JW5ACXX_R2.fastq.gz
Sample_RH41Bromosporinlow_T_C5JW5ACXX_R1.fastq.gz
Sample_RH41Bromosporinlow_T_C5JW5ACXX_R2.fastq.gz
Sample_RH41Cpd50high_T_C4JM1ACXX_R1.fastq.gz
Sample_RH41Cpd50high_T_C4JM1ACXX_R2.fastq.gz
Sample_RH41Cpd50low_T_C4JM1ACXX_R1.fastq.gz
Sample_RH41Cpd50low_T_C4JM1ACXX_R2.fastq.gz
Sample_RH41DMSOhigh_T_C4JM1ACXX_R1.fastq.gz
Sample_RH41DMSOhigh_T_C4JM1ACXX_R2.fastq.gz
Sample_RH41DMSOhigh_T_H144EBGXX_R1.fastq.gz
Sample_RH41DMSOhigh_T_H144EBGXX_R2.fastq.gz
Sample_RH41DMSOlow_T_C4JM1ACXX_R1.fastq.gz
Sample_RH41DMSOlow_T_C4JM1ACXX_R2.fastq.gz
Sample_RH41DMSOlow_T_H144EBGXX_R1.fastq.gz
Sample_RH41DMSOlow_T_H144EBGXX_R2.fastq.gz
Sample_RH41EZ-005high_T_C5JW5ACXX_R1.fastq.gz
Sample_RH41EZ-005high_T_C5JW5ACXX_R2.fastq.gz
Sample_RH41EZ-005low_T_C5JW5ACXX_R1.fastq.gz
Sample_RH41EZ-005low_T_C5JW5ACXX_R2.fastq.gz
Sample_RH41GSKJ4high_T_C4JM1ACXX_R1.fastq.gz
Sample_RH41GSKJ4high_T_C4JM1ACXX_R2.fastq.gz
Sample_RH41GSKJ4low_T_C4JM1ACXX_R1.fastq.gz
Sample_RH41GSKJ4low_T_C4JM1ACXX_R2.fastq.gz
Sample_RH41GSKLSD1high_T_H0VWNAGXX_R1.fastq.gz
Sample_RH41GSKLSD1high_T_H0VWNAGXX_R2.fastq.gz
Sample_RH41GSKLSD1low_T_H0VWNAGXX_R1.fastq.gz
Sample_RH41GSKLSD1low_T_H0VWNAGXX_R2.fastq.gz
Sample_RH41JQ1high_T_C4JM1ACXX_R1.fastq.gz
Sample_RH41JQ1high_T_C4JM1ACXX_R2.fastq.gz
Sample_RH41JQ1low_T_C4JM1ACXX_R1.fastq.gz
Sample_RH41JQ1low_T_C4JM1ACXX_R2.fastq.gz
Sample_RH41LAQ824high_T_H144EBGXX_R1.fastq.gz
Sample_RH41LAQ824high_T_H144EBGXX_R2.fastq.gz
Sample_RH41LAQ824low_T_H144EBGXX_R1.fastq.gz
Sample_RH41LAQ824low_T_H144EBGXX_R2.fastq.gz
Sample_RH41LSD519high_T_C5JW5ACXX_R1.fastq.gz
Sample_RH41LSD519high_T_C5JW5ACXX_R2.fastq.gz

Sample_RH41LSD519low_T_C5JW5ACXX_R1.fastq.gz
Sample_RH41LSD519low_T_C5JW5ACXX_R2.fastq.gz
Sample_RH41LSD690high_T_C5JW5ACXX_R1.fastq.gz
Sample_RH41LSD690high_T_C5JW5ACXX_R2.fastq.gz
Sample_RH41LSD690low_T_C5JW5ACXX_R1.fastq.gz
Sample_RH41LSD690low_T_C5JW5ACXX_R2.fastq.gz
Sample_RH41MC1568high_T_H144EBGXX_R1.fastq.gz
Sample_RH41MC1568high_T_H144EBGXX_R2.fastq.gz
Sample_RH41MC1568low_T_H144EBGXX_R1.fastq.gz
Sample_RH41MC1568low_T_H144EBGXX_R2.fastq.gz
Sample_RH41Merck60high_T_C5JW5ACXX_R1.fastq.gz
Sample_RH41Merck60high_T_C5JW5ACXX_R2.fastq.gz
Sample_RH41Merck60low_T_C5JW5ACXX_R1.fastq.gz
Sample_RH41Merck60low_T_C5JW5ACXX_R2.fastq.gz
Sample_RH41OJI1high_T_C5JW5ACXX_R1.fastq.gz
Sample_RH41OJI1high_T_C5JW5ACXX_R2.fastq.gz
Sample_RH41OJI1low_T_C5JW5ACXX_R1.fastq.gz
Sample_RH41OJI1low_T_C5JW5ACXX_R2.fastq.gz
Sample_RH41PFI2high_T_C5JW5ACXX_R1.fastq.gz
Sample_RH41PFI2high_T_C5JW5ACXX_R2.fastq.gz
Sample_RH41PFI2low_T_C5JW5ACXX_R1.fastq.gz
Sample_RH41PFI2low_T_C5JW5ACXX_R2.fastq.gz
Sample_RH41PFI3high_T_C5JW5ACXX_R1.fastq.gz
Sample_RH41PFI3high_T_C5JW5ACXX_R2.fastq.gz
Sample_RH41PFI3low_T_C5JW5ACXX_R1.fastq.gz
Sample_RH41PFI3low_T_C5JW5ACXX_R2.fastq.gz
Sample_RH41SGC0946high_T_C5JW5ACXX_R1.fastq.gz
Sample_RH41SGC0946high_T_C5JW5ACXX_R2.fastq.gz
Sample_RH41SGC0946low_T_C5JW5ACXX_R1.fastq.gz
Sample_RH41SGC0946low_T_C5JW5ACXX_R2.fastq.gz
Sample_RH41SGCCBP30high_T_C5JW5ACXX_R1.fastq.gz
Sample_RH41SGCCBP30high_T_C5JW5ACXX_R2.fastq.gz
Sample_RH41SGCCBP30low_T_C5JW5ACXX_R1.fastq.gz
Sample_RH41SGCCBP30low_T_C5JW5ACXX_R2.fastq.gz
Sample_RH41Tramhigh_T_C5JW5ACXX_R1.fastq.gz
Sample_RH41Tramhigh_T_C5JW5ACXX_R2.fastq.gz
Sample_RH41Tramlow_T_C5JW5ACXX_R1.fastq.gz
Sample_RH41Tramlow_T_C5JW5ACXX_R2.fastq.gz
Sample_RH41UNC0638high_T_C5JW5ACXX_R1.fastq.gz
Sample_RH41UNC0638high_T_C5JW5ACXX_R2.fastq.gz
Sample_RH41UNC0638low_T_C5JW5ACXX_R1.fastq.gz
Sample_RH41UNC0638low_T_C5JW5ACXX_R2.fastq.gz
Sample_RH41UNC1215high_T_H0VWNAGXX_R1.fastq.gz
Sample_RH41UNC1215high_T_H0VWNAGXX_R2.fastq.gz
Sample_RH41UNC1215low_T_H0VWNAGXX_R1.fastq.gz
Sample_RH41UNC1215low_T_H0VWNAGXX_R2.fastq.gz
Sample_RH41UNC1999high_T_C5JW5ACXX_R1.fastq.gz
Sample_RH41UNC1999high_T_C5JW5ACXX_R2.fastq.gz
Sample_RH41UNC1999low_T_C5JW5ACXX_R1.fastq.gz
Sample_RH41UNC1999low_T_C5JW5ACXX_R2.fastq.gz
Sample_RH41WT161high_T_C5JW5ACXX_R1.fastq.gz
Sample_RH41WT161high_T_C5JW5ACXX_R2.fastq.gz
Sample_RH41WT161low_T_C5JW5ACXX_R1.fastq.gz
Sample_RH41WT161low_T_C5JW5ACXX_R2.fastq.gz
Sample_RH4_D1_T_HVNVFBGX2_R1.fastq.gz
Sample_RH4_D1_T_HVNVFBGX2_R2.fastq.gz
Sample_RH4_D24_T_HVNVFBGX2_R1.fastq.gz
Sample_RH4_D24_T_HVNVFBGX2_R2.fastq.gz
Sample_RH4_D6_T_HVNVFBGX2_R1.fastq.gz
Sample_RH4_D6_T_HVNVFBGX2_R2.fastq.gz
Sample_Rh4_DMSO_T_HCK52BGXY_R1.fastq.gz
Sample_Rh4_DMSO_T_HCK52BGXY_R2.fastq.gz
Sample_RH4_ENT1_T_HVNVFBGX2_R1.fastq.gz
Sample_RH4_ENT1_T_HVNVFBGX2_R2.fastq.gz
Sample_RH4_ENT24_T_HVNVFBGX2_R1.fastq.gz
Sample_RH4_ENT24_T_HVNVFBGX2_R2.fastq.gz
Sample_RH4_ENT6_T_HVNVFBGX2_R1.fastq.gz
Sample_RH4_ENT6_T_HVNVFBGX2_R2.fastq.gz
Sample_Rh4_N1302_T_HCK52BGXY_R1.fastq.gz
Sample_Rh4_N1302_T_HCK52BGXY_R2.fastq.gz
Sample_Rh4_PLX1_T_HCK52BGXY_R1.fastq.gz
Sample_Rh4_PLX1_T_HCK52BGXY_R2.fastq.gz
Sample_Rh4_PLX2_T_HCK52BGXY_R1.fastq.gz
Sample_Rh4_PLX2_T_HCK52BGXY_R2.fastq.gz

ChIP-seq:
Sample_RH4_HDAC2_005_C_H7WKVBGXX_peaks.narrowPeak.nobl.GREAT.bed
Sample_RH4_SOX8_016_C_HJ73HBGXY_peaks.narrowPeak.nobl.GREAT.bed
Sample_RH3_H3K27ac_016_C_HJ73HBGXY_peaks.narrowPeak.nobl.GREAT.bed
Sample_RH4_D6_bioJQ1_016_C_HJ73HBGXY_peaks.narrowPeak.nobl.GREAT.bed
Sample_RH4_D6_H3K27ac_018_C_HWC77BGXY_peaks.narrowPeak.nobl.GREAT.bed
Sample_RH4_Ent1_H3K27ac_018_C_HWC77BGXY_peaks.narrowPeak.nobl.GREAT.bed
Sample_RH4_Ent6_H3K27ac_018_C_HWC77BGXY_peaks.narrowPeak.nobl.GREAT.bed
Sample_RH4_D6_Pol2_018_C_HWC77BGXY_peaks.narrowPeak.nobl.GREAT.bed
Sample_RH4_Ent1_Pol2_018_C_HWC77BGXY_peaks.narrowPeak.nobl.GREAT.bed
Sample_RH4_Ent6_Pol2_018_C_HWC77BGXY_peaks.narrowPeak.nobl.GREAT.bed
Sample_RH30_H3K27ac_016_C_H5JKVBGX3_peaks.narrowPeak.nobl.GREAT.bed
Sample_RH4_D6_H3K36ac_021_C_H5JKVBGX3_peaks.narrowPeak.nobl.GREAT.bed
Sample_RH4_Ent6_H3K36ac_021_C_H5JKVBGX3_peaks.narrowPeak.nobl.GREAT.bed
Sample_RH4_D6_RAD21_021_C_H5JKVBGX3_peaks.narrowPeak.nobl.GREAT.bed
Sample_RH4_Ent6_RAD21_021_C_H5JKVBGX3_peaks.narrowPeak.nobl.GREAT.bed
Sample_RH4_D6_HDAC2_021_C_H5JKVBGX3_peaks.narrowPeak.nobl.GREAT.bed
Sample_RH4_Ent6_HDAC2_021_C_H5JKVBGX3_peaks.narrowPeak.nobl.GREAT.bed
Sample_RH4_D6_HDAC1_024_C_HLFMLBGX3_peaks.narrowPeak.nobl.GREAT.bed
Sample_RH4_D6_RAD21_025_C_HFH72BGX5_peaks.narrowPeak.nobl.GREAT.bed
Sample_RH4_Ent6_RAD21_025_C_HFH72BGX5_peaks.narrowPeak.nobl.GREAT.bed
Sample_RH4_D6_YY1_025_C_HFH72BGX5_peaks.narrowPeak.nobl.GREAT.bed
Sample_RH4_Ent6_YY1_025_C_HFH72BGX5_peaks.narrowPeak.nobl.GREAT.bed
Sample_RH4_DMSO_6hr_ATAC_H7H5CBGX5_peaks.narrowPeak.nobl.GREAT.bed
Sample_RH4_Ent_1hr_ATAC_H7H5CBGX5_peaks.narrowPeak.nobl.GREAT.bed
Sample_RH4_Ent_6hr_ATAC_H7H5CBGX5_peaks.narrowPeak.nobl.GREAT.bed
Sample_RH4_HDAC2_005_C_H7WKVBGXX_R1.fastq.gz
Sample_RH4_SOX8_016_C_HJ73HBGXY_R1.fastq.gz
Sample_RH3_H3K27ac_016_C_HJ73HBGXY_R1.fastq.gz
Sample_RH3_input_016_C_HJ73HBGXY_R1.fastq.gz
Sample_RH4_D6_bioJQ1_016_C_HJ73HBGXY_R1.fastq.gz
Sample_RH4_D6_H3K27ac_018_C_HWC77BGXY_R1.fastq.gz
Sample_RH4_Ent1_H3K27ac_018_C_HWC77BGXY_R1.fastq.gz
Sample_RH4_Ent6_H3K27ac_018_C_HWC77BGXY_R1.fastq.gz
Sample_RH4_D6_Pol2_018_C_HWC77BGXY_R1.fastq.gz
Sample_RH4_Ent1_Pol2_018_C_HWC77BGXY_R1.fastq.gz
Sample_RH4_Ent6_Pol2_018_C_HWC77BGXY_R1.fastq.gz
Sample_RH30_H3K27ac_016_C_H5JKVBGX3_R1.fastq.gz
Sample_RH30_input_016_C_H5JKVBGX3_R1.fastq.gz
Sample_RH4_D6_H3K36ac_021_C_H5JKVBGX3_R1.fastq.gz
Sample_RH4_Ent6_H3K36ac_021_C_H5JKVBGX3_R1.fastq.gz
Sample_RH4_D6_RAD21_021_C_H5JKVBGX3_R1.fastq.gz
Sample_RH4_Ent6_RAD21_021_C_H5JKVBGX3_R1.fastq.gz
Sample_RH4_D6_HDAC2_021_C_H5JKVBGX3_R1.fastq.gz
Sample_RH4_Ent6_HDAC2_021_C_H5JKVBGX3_R1.fastq.gz
Sample_RH4_D6_HDAC1_024_C_HLFMLBGX3_R1.fastq.gz
Sample_RH4_D6_RAD21_025_C_HFH72BGX5_R1.fastq.gz
Sample_RH4_Ent6_RAD21_025_C_HFH72BGX5_R1.fastq.gz
Sample_RH4_D6_YY1_025_C_HFH72BGX5_R1.fastq.gz
Sample_RH4_Ent6_YY1_025_C_HFH72BGX5_R1.fastq.gz
Sample_RH4_DMSO_6hr_ATAC_H7H5CBGX5_R1.fastq.gz
Sample_RH4_Ent_1hr_ATAC_H7H5CBGX5_R1.fastq.gz
Sample_RH4_Ent_6hr_ATAC_H7H5CBGX5_R1.fastq.gz
Sample_RH4_DMSO_6hr_ATAC_H7H5CBGX5_R2.fastq.gz
Sample_RH4_Ent_1hr_ATAC_H7H5CBGX5_R2.fastq.gz
Sample_RH4_Ent_6hr_ATAC_H7H5CBGX5_R2.fastq.gz

Genome browser session
(e.g. UCSC)

We viewed all datasets in IGV, not a public browser website. TDF files, a compressed version of BEDGRAPHS for IGV viewing, can be made available to reviewers if needed.

Methodology

Replicates

ChIP-seq replicates were performed across multiple cell lines rather than multiple experiments per cell line, with the exception of RAD21 in RH4 cells, which was performed in biological duplicate (deposited as Rep1 and Rep2).

Sequencing depth

All ChIP-seq and ChIP-Rx experiments were performed in single-end read mode, 75 base pairs. Spike in reads were measured in parrallel mapping to dm3 and hg19, with results as follows:
ChIP-Rx Sample name Human_Reads
Sample_RH4_D6_BRD4_024_C_HLFMLBGX3 33045619
Sample_RH4_D6_HDAC2_021_C_H5JKVBGX3 23460819
Sample_RH4_D6_p300_024_C_HLFMLBGX3 26822752
Sample_RH4_D6_RAD21_021_C_H5JKVBGX3 16714974
Sample_RH4_D6_RAD21_025_C_HFH72BGX5 51895856
Sample_RH4_D6_YY1_025_C_HFH72BGX5 23373134
Sample_RH4_Ent6_BRD4_024_C_HLFMLBGX3 72847125

Sample_RH4_Ent6_HDAC2_021_C_H5JKVBGX3 24487174
Sample_RH4_Ent6_p300_024_C_HLFMLBGX3 22568713
Sample_RH4_Ent6_RAD21_021_C_H5JKVBGX3 22368744
Sample_RH4_Ent6_RAD21_025_C_HFH72BGX5 37802129
Sample_RH4_Ent6_YY1_025_C_HFH72BGX5 40957167
Sample_RH4_D6_H3K27ac_018_C_HWC77BGXY 29336224
Sample_RH4_D6_Pol2_018_C_HWC77BGXY 45354877
Sample_RH4_Ent1_H3K27ac_018_C_HWC77BGXY 31486542
Sample_RH4_Ent1_Pol2_018_C_HWC77BGXY 33191147
Sample_RH4_Ent6_H3K27ac_018_C_HWC77BGXY 38211649
Sample_RH4_Ent6_Pol2_018_C_HWC77BGXY 35643654
Sample_RH4_D6_H3K36ac_021_C_H5JKVBGX3 29947004
Sample_RH4_Ent6_H3K36ac_021_C_H5JKVBGX3 22926282

ChIP-Rx Sample name SpikeIn_Drosophila_Reads
Sample_RH4_D6_BRD4_024_C_HLFMLBGX3 1072779
Sample_RH4_D6_HDAC2_021_C_H5JKVBGX3 423584
Sample_RH4_D6_p300_024_C_HLFMLBGX3 3786163
Sample_RH4_D6_RAD21_021_C_H5JKVBGX3 949948
Sample_RH4_D6_RAD21_025_C_HFH72BGX5 813980
Sample_RH4_D6_YY1_025_C_HFH72BGX5 659906
Sample_RH4_Ent6_BRD4_024_C_HLFMLBGX3 2023651
Sample_RH4_Ent6_HDAC2_021_C_H5JKVBGX3 606145
Sample_RH4_Ent6_p300_024_C_HLFMLBGX3 4025804
Sample_RH4_Ent6_RAD21_021_C_H5JKVBGX3 1263186
Sample_RH4_Ent6_RAD21_025_C_HFH72BGX5 414133
Sample_RH4_Ent6_YY1_025_C_HFH72BGX5 781349
Sample_RH4_D6_H3K27ac_018_C_HWC77BGXY 650454
Sample_RH4_D6_Pol2_018_C_HWC77BGXY 3661245
Sample_RH4_Ent1_H3K27ac_018_C_HWC77BGXY 663539
Sample_RH4_Ent1_Pol2_018_C_HWC77BGXY 2647315
Sample_RH4_Ent6_H3K27ac_018_C_HWC77BGXY 586269
Sample_RH4_Ent6_Pol2_018_C_HWC77BGXY 3687619
Sample_RH4_D6_H3K36ac_021_C_H5JKVBGX3 694793
Sample_RH4_Ent6_H3K36ac_021_C_H5JKVBGX3 373765

Antibodies

same antibodies listed above.

Peak calling parameters

Peaks were called using MACS2 (version 2.1.1.20160309, https://github.com/taoliu/MACS) using "narrow" mode for all targets reported in this paper, as they form sharp genomic peaks. Parameters for MACS2 usage: [--format BAM --control input.bam --keep-dup all --pvalue 0.0000001]. Regions called as peaks which are known to be spurious mapping artifacts were removed before any further analysis (reference locations for sites black-listed by the ENCODE consortium, https://sites.google.com/site/anshulkundaje/projects/blacklists).

Data quality

Samples are listed with the number of peaks at a threshold of p < 1x10^(-7) in the 2nd column, and, in the 3rd column, the number of peaks with at least 5-fold or more enrichment are reported.
SampleFile PEAKS_PVALUE7 PEAKS_FOLD_CHANGE5
Sample_RH4_H3K27ac_001_C_H5TLGBGXX 69895 66719
Sample_RH4_HDAC2_005_C_H7WKVBGXX 31422 29444
Sample_SCMC_H3K27ac_008_C_HHC7JBGXX 39084 38125
Sample_RH5_H3K27ac_008_C_HHC7JBGXX 37036 38696
Sample_RD_H3K27ac_008_C_HHC7JBGXX 41078 38039
Sample_RH18_H3K27ac_009_C_HHC7KBGXX 58143 54262
Sample_CTR_D48_H3K27ac_010_C_HMKGLBGXX 38141 36786
Sample_CTR_T48_H3K27ac_010_C_HMKGLBGXX 33382 33273
Sample_RH4_SOX8_016_C_HJ73HBGXY 5190 5069
Sample_RH3_H3K27ac_016_C_HJ73HBGXY 40904 35683
Sample_RH3_P3F_016_C_HJ73HBGXY 4609 4365
Sample_RH4_D6_bioJQ1_016_C_HJ73HBGXY 21905 20612
Sample_RMS559_H3K27ac_016_C_HJ73HBGXY 54360 41203
Sample_RMS238_H3K27ac_007_C_HHF57BGXX 4988 3107
Sample_RMS209_H3K27ac_007_C_HHF57BGXX 31464 33674
Sample_NCI0082_H3K27ac_007_C_HHF57BGXX 29736 28428
Sample_RMS206_H3K27ac_007_C_HHF57BGXX 28789 30773
Sample_RMS216_H3K27ac_007_C_HHF57BGXX 33656 34696
Sample_RMS008_H3K27ac_007_C_HHF57BGXX 32943 31325
Sample_NCI0075_H3K27ac_007_C_HHF57BGXX 23894 25122
Sample_RH4_D6_H3K27ac_018_C_HWC77BGXY 62043 58677
Sample_RH4_Ent1_H3K27ac_018_C_HWC77BGXY 63599 60301
Sample_RH4_Ent6_H3K27ac_018_C_HWC77BGXY 71043 64212
Sample_RH4_D6_Pol2_018_C_HWC77BGXY 27014 24297
Sample_RH4_Ent1_Pol2_018_C_HWC77BGXY 21338 19232

Sample_RH4_Ent6_Pol2_018_C_HWC77BGXY 29228 26854
Sample_Birch_H3K27ac_016_C_H5JKVBGX3 27818 27711
Sample_RH30_H3K27ac_016_C_H5JKVBGX3 37404 36761
Sample_RH4_D6_H3K36ac_021_C_H5JKVBGX3 21309 20147
Sample_RH4_Ent6_H3K36ac_021_C_H5JKVBGX3 13744 13319
Sample_RH4_D6_RAD21_021_C_H5JKVBGX3 13350 13351
Sample_RH4_Ent6_RAD21_021_C_H5JKVBGX3 21278 21256
Sample_RH4_D6_HDAC2_021_C_H5JKVBGX3 14590 14266
Sample_RH4_Ent6_HDAC2_021_C_H5JKVBGX3 28588 27817
Sample_RH4_D6_HDAC1_024_C_HLFMLBGX3 75609 72600
Sample_RH4_D6_RAD21_025_C_HFH72BGX5 37633 36978
Sample_RH4_Ent6_RAD21_025_C_HFH72BGX5 39160 38543
Sample_RH4_D6_YY1_025_C_HFH72BGX5 24398 23081
Sample_RH4_Ent6_YY1_025_C_HFH72BGX5 18963 17921
Sample_RH4_DMSO_6hr_ATAC_H7H5CBGX5 15440 13650
Sample_RH4_Ent_1hr_ATAC_H7H5CBGX5 29204 27063
Sample_RH4_Ent_6hr_ATAC_H7H5CBGX5 49207 45234

Software

Motif analysis was performed on peaks called from MACS2, using findMotifsGenome.pl from HOMER version 4.9.1 (http://homer.ucsd.edu/homer/index.html). Super enhancers were identified using the ROSE2 package (https://github.com/linlabbcm/rose2) employing stitching parameter of 12500 bp. The scripts used for core regulatory analysis are available here (https://pypi.org/project/coltron/). For peak comparisons, we used Bedtools version 2.27.1 (http://bedtools.readthedocs.io/en/latest/). Plots of ChIP-seq data were made with NGSplot v. 2.63 (https://github.com/shenlab-sinai/ngsplot).
